# Determination of Starch Size Distribution in Single Grains (SSDSG): A Novel High-Efficiency Method for Characterizing Starch Granule Morphology in Wheat

**DOI:** 10.3390/foods15213971

**Published:** 2026-11-05

**Authors:** Xiaoyan Duan, Shuai Yan, Lei Guo, Aifeng Liu, Yifei Chen, Danping Li, Wenjia Zhang, Canguo Wang, Yirui Wang, Xiaojun Nie, Jianjun Liu, Zhendong Zhao, Jinpeng Zou, Yuelan Liu, Shengnan Zhai, Xinyou Cao, Dungong Cheng, Xin Gao

**Affiliations:** 1 Crop Research Institute, Shandong Academy of Agricultural Sciences/National Engineering Research Center of Wheat and Maize/State Key Laboratory of Wheat Improvement/Key Laboratory of Wheat Biology and Genetic Improvement in North Yellow & Huai River Valley, Shandong Provincial Technology Innovation Center for Wheat, Jinan 250100, China; 2 College of Agronomy, Northwest A&F University, Yangling 712100, China

**Keywords:** wheat (*Triticum aestivum* L.), grain quality, starch granule size distribution, SSDSG, single-grain phenotyping, high-throughput methodology

## Abstract

Wheat starch granule size distribution is a critical determinant of processing quality and end-use functionality. However, conventional methods for characterizing starch granules are labor-intensive, require relatively large sample quantities, and may preferentially lose small B-type granules during starch extraction. To address these limitations, we developed SSDSG (Determination of Starch Size Distribution in Single Grains), a novel, high-throughput, image-based method for in situ and non-destructive phenotyping of starch granule size distribution at the single grain level. A morphological correction factor ($\tilde{K}=0.4113$) enabled statistical conversion to volume equivalent measurements, and the method showed a significant positive correlation with the Coulter counter reference method (*R*^2^ = 0.55) across 20 wheat varieties. SSDSG characterized the temporal dynamics during grain filling and intra-spike spatial heterogeneity, providing technical support for dissecting grain development and analyzing superior and inferior grains. High-throughput screening of 1667 EMS-mutagenized lines revealed broad variation in B-type starch proportion in total starch (84.01–98.69%), which is significant for further genetics and phenotypic analysis. Moreover, the non-destructive workflow allows a single grain being used for both phenotyping and propagation. The top 10% of the 204 F_2_ breeding lines, with highest B-type starch proportion, were selected and propagated for further genetic analysis. Finally, characterization of barley and 24 diverse-ploidy wheat accessions revealed that tetraploid wheat had relatively lower B-type starch proportions. This is significant for understanding starch traits evolution during wheat polyploidization. Overall, SSDSG provides a practical high-throughput platform for single-grain starch phenotyping and quality-oriented wheat breeding.

## 1. Introduction

Wheat is a major staple crop and plays a central role in global food security [1]. Its end-use quality is intrinsically linked to the physicochemical properties of starch [2,3], which constitutes the predominant component of the grain [4,5]. Among these properties, starch granule morphology and size distribution are important determinants of wheat processing performance [6,7,8]. In this study, starch granules were classified into A-type (>10 μm) and B-type (≤10 μm) granules according to diameter [9]. Recent studies have shown that a moderate increase in the proportion of B-type granules [2] can positively influence both end-use processing quality and nutritional characteristics [10,11,12], partly by strengthening starch–gluten interactions and gluten network continuity. For example, an A-/B-type ratio of approximately 75: 25 improved bread dough extensibility and specific volume, and 5% B-type granule addition increased SDS-unextractable polymeric protein and bound water content, whereas excessive B-type granules can impair gluten hydration and dough performance [13,14]. However, substantial variation exists among individual wheat kernels, and conventional bulk-scale analyses may obscure this grain-to-grain heterogeneity [15]. Therefore, the development of a rapid, practical, and high-throughput method for characterizing starch granule size distribution at the single-grain level is essential for more precise wheat breeding, quality evaluation, and quality control.

Conventional techniques for starch granule size analysis, including Coulter counting (electrical sensing zone method), laser diffraction, and scanning electron microscopy (SEM) [16], are widely employed but have several inherent limitations [17,18]. All these approaches require a critical step: starch extraction [19]. There are two commonly adopted starch extraction methods: wet separation and dry separation. As a mainstream conventional industrial technique, wet separation enables the processing of large-volume samples. Nevertheless, it not only suffers from relatively high energy consumption, but also leads to the loss of partial fine B-type starch granules during sedimentation. This loss is quantitatively significant: during laboratory fractionation, 7.6–11.6% of the total starch remains unrecovered, and notably, the fraction yields of B-type starch granules are substantially lower than their established contents in the respective native starches [20]. This selective loss of small granules alters the composition of the recovered starch and distorts its characteristic bimodal size distribution [21], thereby directly compromising the reliability of size-distribution measurements based on extracted starch [22]. Moreover, it is a time-consuming separation method, the subsequent extracts requiring prolonged drying, which restricts its applicability for research-oriented demands. By contrast, dry separation features low energy consumption and mild extraction conditions, which can well preserve the native structure of starch [23]. However, powder agglomeration frequently occurs, impairing the accuracy of subsequent starch fractionation, which makes the entire procedure time-consuming and labor-intensive [19]. Regardless of the extraction method, approximately 6 g of wheat grains is generally required to acquire reliable results for starch particle-size measurement [24,25]. This relatively large sample requirement limits the application of these methods to high-throughput screening and the analysis of limited germplasm resources, while the resulting bulk analysis inherently masks individual grain-to-grain variations. In addition, laser diffraction may underestimate the diameter of A-type granules because particle size is estimated from light-scattering patterns, potentially resulting in systematic measurement errors [18].

The above disadvantages of starch extraction also affect the accuracy of most of the existing image-based methods, which use the image data derived from the extracted starch samples for further qualitative or semi-quantitative morphological assessment, resulting in limited capacity for robust quantitative and high-throughput analysis [26]. In recent years, single-grain studies have attracted increasing attention in wheat quality research. For example, a recent study utilized single-seed hyperspectral imaging to quantify grain quality traits across diverse wheat landraces, effectively resolving grain-to-grain heterogeneity. This approach not only achieved highly accurate predictions of grain protein content (GPC) and grain weight (GW) but also enabled the genetic dissection of these traits [27]. However, high-throughput, embryo-preserving, and non-destructive analysis of starch granule size distribution at the single-grain level remains less established.

To address these limitations, we developed a novel high-throughput method for determining starch size distribution in single grains (SSDSG). The workflow integrates single-grain preparation, microscopic imaging, and batch data analysis via custom-developed HTDP software (Version 1.0). This streamlined process eliminates the need for labor-intensive flour milling and starch extraction, thereby minimizing the loss of B-type granules. Furthermore, it enables rapid, non-destructive, in situ, and high-throughput characterization of starch granules at the single-grain level. In addition, only a single grain is required for each measurement, substantially reducing sample consumption and making the method particularly suitable for the analysis of limited or valuable genetic resources. The proprietary software enables rapid data processing and automated generation of starch size distribution parameters, markedly reducing analysis time while improving analytical efficiency. Beyond method development, SSDSG provides analysis platform for dissecting starch granule formation during grain development by monitoring grain-filling dynamics and analyzing superior/inferior grains by distinguishing intra-spike spatial heterogeneity. It also enables high-throughput screening of EMS-mutagenized populations, revealing broad variation that is significant for future genetic analysis. Furthermore, its non-destructive approach enables simultaneous phenotyping and propagation, facilitating early-generation selection and genetic stability evaluation, while its application across diverse cereal species and wheat ploidy levels offers insights into the evolution of starch traits during polyploidization. These features establish SSDSG as a practical, high-throughput platform for accelerating starch-related genetic research and quality-oriented wheat breeding.

## 2. Materials and Methods

### 2.1. Materials

The primary research object of this study is wheat. Twenty wheat varieties were used to validate the SSDSG method and assess its correlation with conventional Coulter counter measurements. These varieties are cultivars or breeding lines widely grown in Huang-Huai region of China or used as crucial parental materials in wheat breeding in the relative breeding institutions. They were randomly selected to cover a relatively wide range of variation in starch size distribution. These varieties included Nongda8987, Qifeng2, Daimai3166, Shannong0431, Zhoumai40, JinongCH01, Xinong979, JimaiU80, Xingmai13, Shannong25, Hengguan35, Hongdi88, Jihe12339, Huaimai35, Yannong187, XumaiDH9, Zhongyu1526, Zhoumai32, Shimai27, and Zhoumai38. Jimai44 and Fielder were used to investigate the dynamics of starch granule development during grain filling and differences in starch granule distribution between superior and inferior grains, respectively. Xichang 69 and its EMS-mutagenized population containing 1667 lines were used for screening and validating the variation in B-type starch content. An F_2_ population containing 204 lines derived from a cross between Jimai44 and Jimai229 was used for the non-destructive characterization of starch size distribution to facilitate generation advancement in early-stage breeding materials.

Additionally, to evaluate the cross-species and cross-ploidy applicability of the method, twenty-seven accessions with diverse ploidy were used in Section 3.5. These included three barley cultivars (Golden Promise (GP), Morex, and Steptoe), five diploid wheat accessions (Mo101, Bo5, *T. urartu*, Y2280 (DWY2280), Y2282 (DWY2282)), eight tetraploid wheat accessions (69A13 (TABIGHA 85I B85), IG 115807, IG 99236, IG 45352, IG 88764, D3, Langdon, D11), and 11 hexaploid wheats classified into three subsections, including four landraces: Chinese Spring (CS), Yangmai (YM), Chengduguangtou (CDGT), and Sanyuehuang (SYH), four modern cultivars: Aikang58 (AK58), Xinong 511 (XN511), Xinong 2000 (XN2000), and Shanmai159 (SM159), and three synthetic hexaploid wheat accessions: Syn773, Syn789, and Syn791.

### 2.2. Reagents

Glycerol, potassium iodide (KI), and iodine (I_2_) (purity ≥ 99%) were obtained from Macklin (Shanghai Macklin Biochemical Co., Ltd., Shanghai, China), while ethanol (purity ≥ 95%) was purchased from China National Pharmaceutical Group Shanghai Co., Ltd. (Shanghai, China). The KI–I_2_ solution was prepared by sequentially dissolving 8 g of KI and 1 g of I_2_ in 100 mL of distilled water. Iodine was added only after the KI had completely dissolved. The resulting solution was transferred to a brown glass bottle and stored at room temperature (25 °C) until use [28].

### 2.3. Instruments

Microscopic observations of wheat starch granules were performed at 200× magnification using an OLYMPUS BX53 microscope (Olympus Corporation, Hachioji, Japan) equipped with an E3ISPM06300KPB electronic eyepiece (Suzhou Jingkai Instrument Equipment Co., Ltd., Suzhou, China). Images were acquired using ImageView software (Version 4.11.21522.20221011) (Nanjing Puliang Optoelectronic Technology Co., Ltd., Nanjing, China). Image processing and data extraction were conducted using the automatic seed examination and thousand grain weight analyzer (Version SG-G) (Hangzhou WSeen Detection Technology Co., Ltd., Hangzhou, China) in combination with custom-built HTDP software. The microstructure of starch granules was observed using scanning electron microscopy (SEM) at 600× magnification (JEOL JSM-6360LV, Akishima, Japan).

### 2.4. Sample Preparation

**Figure 1:**
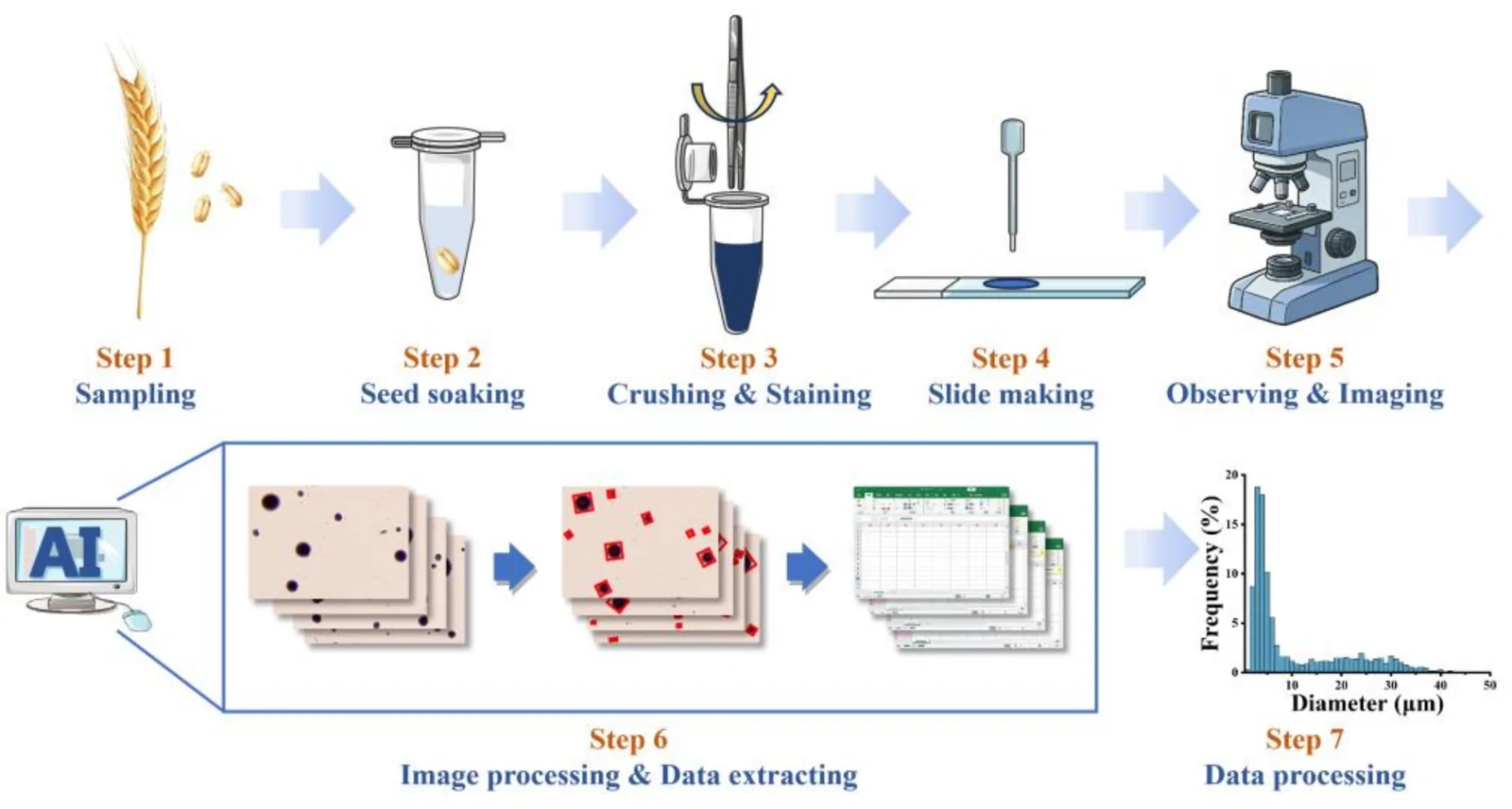
Schematic workflow of the SSDSG (Determination of starch size distribution in single grain) method. Step 1: Sampling of wheat seeds. Step 2: Soaking the selected seeds in water for 48 h. Step 3: Crushing the seeds to release starch granules and staining with I_2_-KI solution. Step 4: Preparation of microscope slides. Step 5: Observation and imaging using an electron microscope. Step 6: Image processing and data extraction assisted by the custom-developed platform. Step 7: Data processing and statistical analysis to obtain final results.

A schematic workflow of the experimental procedure, comprising Steps 1–4, is presented in Figure 1. Except for the experiment examining the effects of the spatial distribution of wheat grains (G1, G2, G3, and G4) on starch size distribution, only superior grains (G1 and G2) in the middle position of a spike were used for all other experiments to minimize potential experimental variation. Briefly, wheat seeds were surface-sterilized with ethanol and subsequently immersed in distilled water for 48 h to ensure complete imbibition. Following hydration, the seed coats were carefully ruptured using forceps to release the endosperm contents. The released materials were treated with 200 μL of potassium iodide–iodine (KI–I_2_) solution, vortexed, and incubated for 1 h to allow adequate staining. Subsequently, 1800 μL of 40% (*v*/*v*) glycerol solution was added, and the mixture was vortexed until homogeneous. Finally, a 10 μL aliquot of the resulting suspension was pipetted onto a glass slide for microscopic observation.

### 2.5. Image Acquisition and Processing

The image acquisition procedure corresponds to Step 5 in Figure 1. Prepared slides were examined using a microscope equipped with a 10× ocular lens and a 20× objective lens. Prior to image acquisition, the imaging system was calibrated at the selected magnification using a stage micrometer to ensure accurate size measurements. Images were captured using ImageView software by randomly selecting multiple fields of view. For each sample, three replicate slides were prepared, and at least ten images were acquired per slide to minimize sampling and experimental variability. For quality control, datasets with a relative standard deviation (RSD) exceeding 5% were not acceptable, so that the images of corresponding sample should be re-captured.

Image processing and data extraction were performed using the automatic seed examination and thousand grain weight analyzer (Hangzhou WSeen Detection Technology Co., Ltd., China). Briefly, the raw image files were imported into the SCG-system, and the corresponding calibration coefficients were applied for size correction. The ‘pennisetum analysis’ module was then selected, and batch processing was initiated for all imported images. Following processing, the system generated two types of outputs: (i) annotated images in which individual starch granules were detected, encircled, and sequentially numbered; and (ii) a corresponding CSV file containing the key morphological parameters of each granule, including diameter, perimeter, and projected area. These data were subsequently used for statistical analysis.

### 2.6. Development of High-Throughput Data Processing Software

This phase corresponds to Steps 6 and 7 in Figure 1. A high-throughput data processing software (HTDP-software) was designed specifically for the analysis of A- and B-type starch granules. The software was developed using Visual C++ in the Microsoft Visual Studio Community 2022 integrated development environment. The high computational efficiency of Visual C++ provides robust support for the rapid processing of starch granule size data, while its advanced file and directory manipulation capabilities facilitate efficient batch processing of large-scale datasets. The developed software automatically performs statistical analyses of starch granule size distributions and generates tabulated results showing the proportional abundance of A- and B-type granules across different size classes. All analytical results are exported in a standardized CSV format, allowing seamless integration with commonly used data analysis and visualization platforms, such as Microsoft Excel and Origin. Users can subsequently import the data into these platforms to generate customized starch granule size distribution curves and other graphical representations. The software is provided in Supplementary Source Code S1.

## 3. Results and Discussion

### 3.1. Method Development: A Novel High-Throughput Quantitative Method for Starch Granule Size Distribution Analysis and Comparison with Conventional Particle-Sizing Techniques

To characterize starch granule size distribution more efficiently, we developed a novel high-throughput imaging-based method, termed the SSDSG method. This approach enables in situ, non-destructive, and quantitative characterization of starch granule size distribution at the single-grain level, thereby overcoming several limitations associated with conventional bulk-based analyses.

The overall workflow of the SSDSG method is schematically illustrated in Figure 1. Briefly, a single wheat grain was collected and soaked in distilled water for 48 h (Steps 1–2). The hydrated grain was then crushed to release the endosperm contents, which were subsequently stained for starch visualization (Step 3). The stained suspension was subsequently transferred onto a glass slide using a pipette (Step 4) and examined by microscopy (Step 5). The acquired raw images were subjected to image preprocessing and morphological feature extraction, followed by automated data extracting and batch analysis using custom-developed HTDP software (Step 6). This integrated workflow enables the characterization of individual starch granules, generating not only measurements of their diameter, perimeter, and projected area, but also statistical distributions of granule counts across predefined size intervals. The resulting comprehensive dataset provides the basis for subsequent quantitative analyses (Step 7).

To evaluate the consistency of the SSDSG method, we compared its performance with that of the Coulter counter, one of the most widely used conventional techniques and a recognized reference method for particle size analysis [11]. However, direct comparison between the two methods is complicated by fundamental differences in their output metrics: the Coulter counter generates volume-based particle size distributions [29], whereas the SSDSG method yields number-based size distributions. To facilitate a meaningful comparison, the number-based SSDSG data were therefore converted into volume-equivalent distributions.

Wheat starch granules exhibit distinct morphologies depending on their size class [11,30]. B-type starch granules are approximately spherical in shape [31], allowing their volumes to be estimated directly from their measured diameters using the spherical volume equation. In contrast, A-type starch granules are characteristically disk-shaped, with their thickness substantially smaller than their equatorial diameter [18,32]. Previous studies have reported that A type starch granules have equatorial diameters ranging from 10 to 40 μm [33], whereas their thickness of 5–15 μm represents a theoretical range derived from the geometric proportions of their disk shaped for volume calculation [18]. Consequently, applying the spherical volume equation to A-type granules would substantially overestimate their actual volume, particularly for larger granules. To account for this morphological difference, we introduced a correction factor $\tilde{K}$, to adjust the estimated volume of A-type granules for their non-spherical geometry.

**Figure 2:**
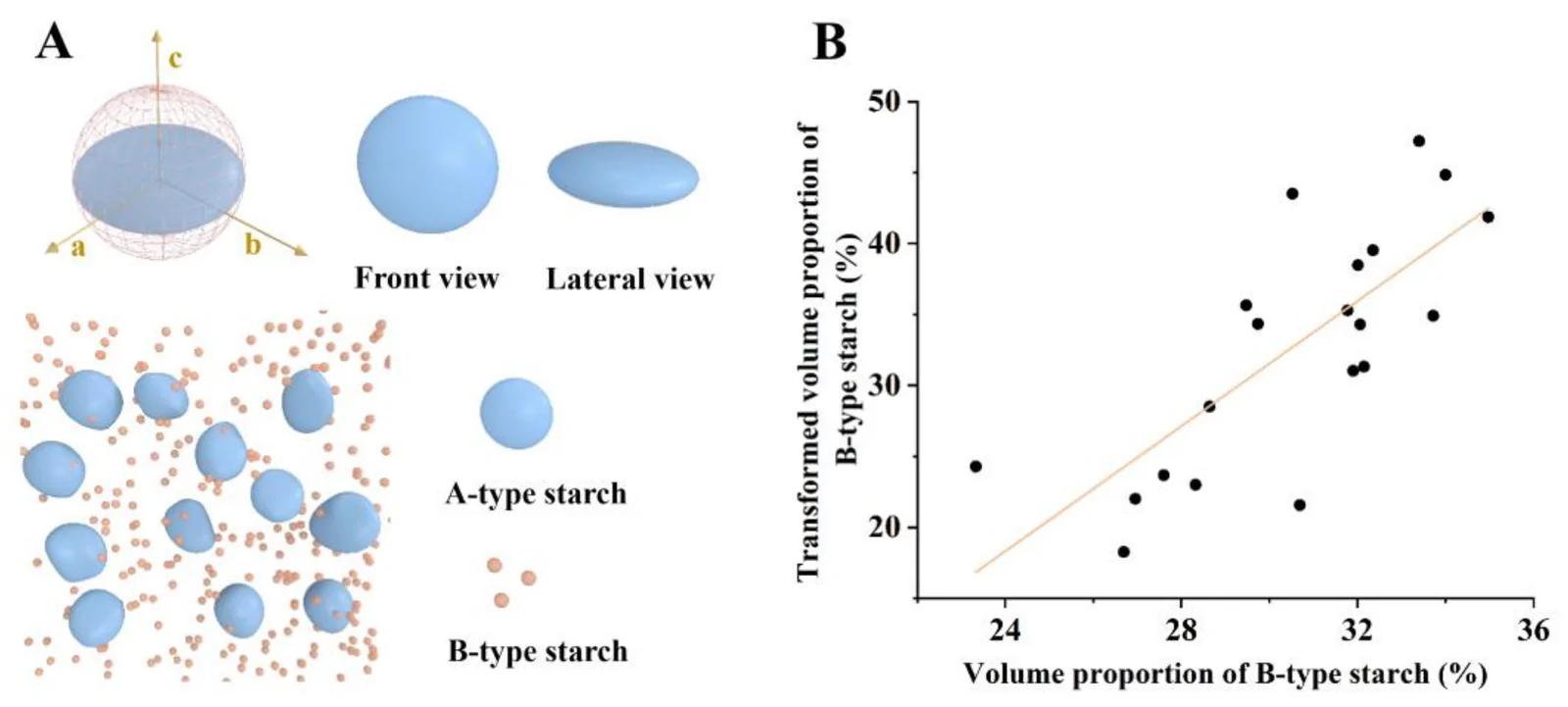
Validation and correlation analysis of the SSDSG (Determination of starch size distribution in single grain) method against conventional Coulter counter measurements. (**A**) Schematic illustration of the oblate spheroid model used for A-type starch granules, indicating equatorial diameter (2a) and thickness (2c). (**B**) Correlation analysis of B-type starch granule volume proportion between the SSDSG method and the Coulter counter across 20 wheat varieties. The orange line represents the linear regression fit (R^2^ = 0.55).

As illustrated in Figure 2A, A-type starch granules were modeled as oblate spheroids, with equatorial diameters (2a) ranging from 10 to 40 μm and thickness (2c) ranging from 5 to 15 μm. Each range was divided into 30 equal intervals: the step size for 2c (a range of 5–15 μm) is 0.3333 μm, and for 2a (a range of 10–40 μm) is 1.0000 μm. A strong linear relationship exists between these parameters: **2a = 6c−5**. The conversion factor K is defined as the ratio of the ellipsoid volume to the equivalent sphere volume:

(1) $$K=\frac{V_{\mathrm{ellipsoid}}}{V_{\mathrm{sphere}}}=\frac{\frac{\pi }{6}{\left(2a\right)}^{2}2c}{\frac{\pi }{6}{\left(2a\right)}^{3}}=\frac{c}{a}$$

Substituting the linear relationship 2a = 6c−5 into this equation, we obtain

(2) $$K=\frac{2c}{6c-5}$$

To construct the corresponding sample sequence, the thickness parameter 2c is set to vary linearly with the index ***i***:

(3) $${2c}_{i}=5+\frac{i}{3}(i=0\sim 30)$$

Integrating these relationships, for each pair (a_i_, c_i_), the correction factor K_i_ was calculated as

(4) $$K_{i}=\frac{c_{i}}{a_{i}}=\frac{5+\frac{i}{3}}{10+i},i=0\sim 30$$

yielding *K_i_* ∈ [0.375, 0.5]. The results indicate that larger A-type starch granules exhibited greater flattening, as reflected by their lower (K) values, consistent with their more pronounced disk-like morphology. To account for this non-spherical morphology in subsequent volume calculations, the average correction factor across the entire A-type granule size range was calculated as

(5) $$\tilde{K}=\frac{1}{31}\sum_{i=0}^{30}K_{i}\approx 0.4113$$

This average correction factor $\tilde{K}$ was then applied to convert the measured circular diameter of individual A-type starch granules into an estimated thickness, thereby enabling subsequent granule volume calculations while accounting for their characteristic flattened morphology. The resulting volume estimates enabled direct, volume-equivalent comparison between the SSDSG method and the Coulter counter method. Detailed calculations are provided in Table S1 and Figure S1.

To assess the consistency and reliability of the SSDSG method, 20 wheat varieties exhibiting contrasting starch properties were randomly selected as test materials. As shown in Figure 2B, the volume proportion of B-type granules determined using the SSDSG method was significantly and positively correlated with the corresponding measurements obtained using the Coulter counter across the 20 wheat varieties (*R*^2^ = 0.55). This significant consistency indicates a general agreement between the two methods, despite their fundamentally different measurement principles (variety-specific comparisons are provided in Table S2).

Collectively, to address the inherent limitations of conventional methods for determining starch granule size distribution, including labor-intensive starch extraction [22], relatively large sample requirements, and the potential loss of B-type granules during purification [25,34], we developed the SSDSG method. And these results demonstrate that, following appropriate morphological correction, the SSDSG method provides the measurements that are broadly comparable with those obtained using the Coulter counter. Importantly, SSDSG substantially reduces sample requirements while enabling high-throughput analysis at the single-grain level. These advantages make the method particularly suitable for large-scale screening of breeding populations and for the analysis of limited or valuable samples.

### 3.2. In Situ Characterization of Starch Granule Size Distribution in Single Wheat Grains

Starch granule size distribution in the wheat endosperm exhibits pronounced spatiotemporal heterogeneity, varying not only across developmental stages during grain filling but also among grains located at different positions within the same spike [35,36]. These spatiotemporal patterns are intrinsically coupled with the regulatory processes governing starch biosynthesis and are therefore important for understanding the molecular mechanisms underlying grain development and quality formation [35,37]. However, conventional approaches typically involve the bulk milling of multiple grains followed by starch extraction, which inevitably averages out inter-grain variation and masks developmental dynamics and positional differences in starch granule characteristics. To overcome these critical limitations, we employed the SSDSG method, which enables high-throughput and quantitative characterization of starch granule size distribution at defined spatial positions in individual grains across developmental stages. This approach allows fine-scale spatial and temporal variations to be resolved, providing information that is largely inaccessible through conventional bulk-sample analyses.

**Figure 3:**
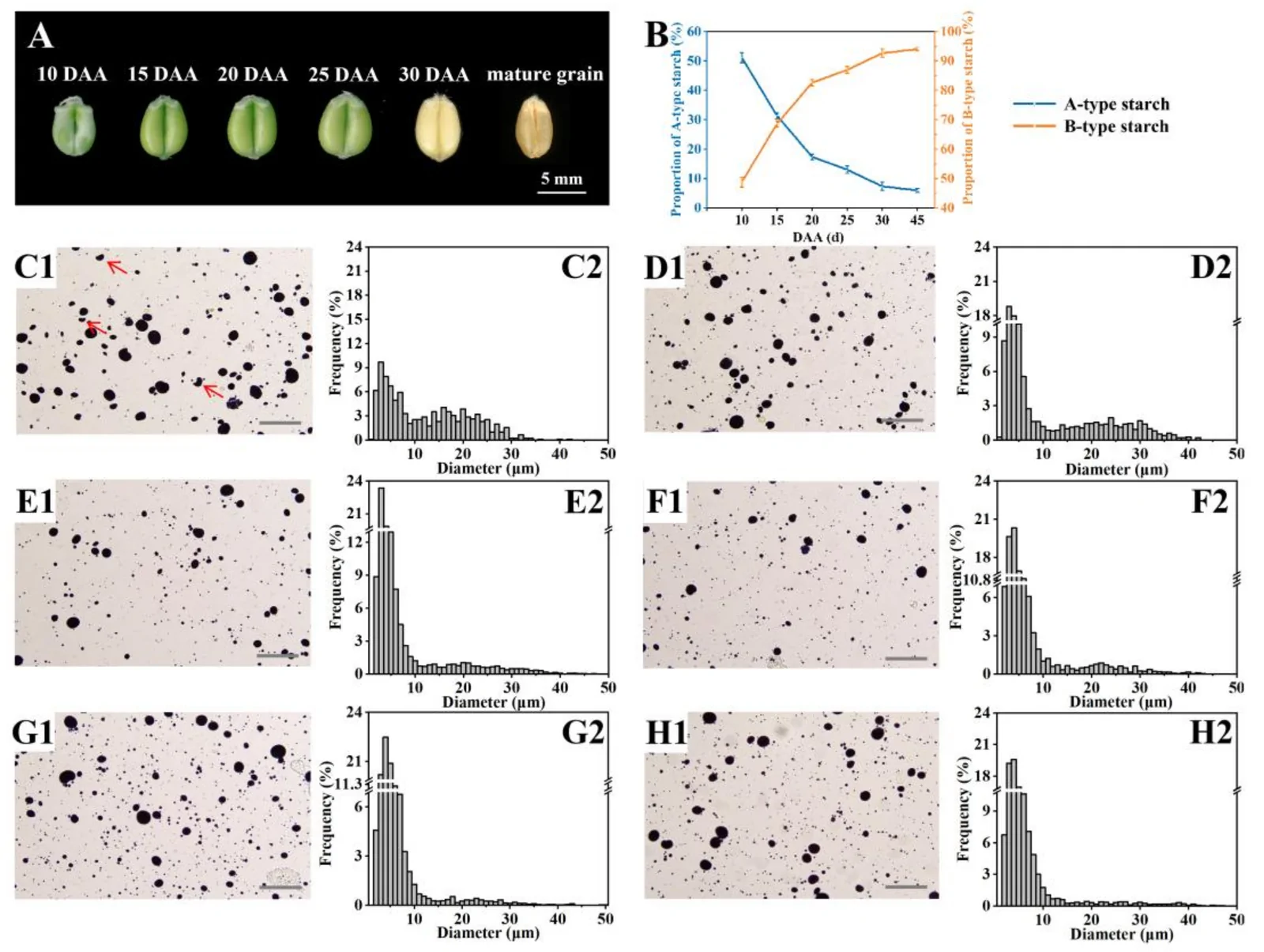
Dynamics of starch granule development during wheat grain filling. (**A**) Morphology of developing grains at different days after anthesis (DAA). Scale bars = 5 mm. (**B**) Temporal changes in the pro-portion of A- and B-type starch granules during grain filling. (**C1**–**H1**) Micrographs showing starch granule size distributions at 10, 15, 20, 25, and 30 DAA, as well as in mature grains, respectively. Scale bars = 50 μm. (**C2**–**H2**) Frequency distribution histograms of starch granule diameters corresponding to the micrographs.

To elucidate starch biosynthesis and accumulation at the single-grain level, we applied the SSDSG method to characterize the development and progression of starch granule formation throughout wheat grain filling (Figure 3A). Starch granule size distributions were assessed at 10, 15, 20, 25, and 30 days after anthesis (DAA), as well as in mature grains (Figure 3B–H). The results revealed a distinct temporal pattern of starch granule development, with A-type granules appearing earlier in grain development, followed by the emergence of B-type granules. Quantification of the relative proportions of the two granule types further demonstrated contrasting developmental trajectories. During starch accumulation, the proportion of A-type starch granules was initially high, gradually declined, and subsequently reached a relatively stable level. In contrast, the proportion of B-type starch granules was initially low but progressively increased before reaching a plateau (Figure 3B). These findings provide direct quantitative evidence that A-type starch granule formation precedes B-type granule initiation. This sequential development is consistent with the established temporal pattern in wheat endosperm, where A-type granules initiate early (approximately 6–8 DAA) [38], and the later stages of grain filling are accompanied by an increase in B-type granule number [39]. This temporal pattern is further confirmed in Figure 3C1, which shows some incompletely developed A-type starch granules at 10 DAA at the positions indicated by the red arrows. Whereas B-type granules begin to form later, at approximately 15–20 DAA [36,40]. Collectively, these temporal patterns reveal the dynamic processes underlying starch biosynthesis and accumulation and provide a quantitative framework for dissecting starch granule development during grain filling.

**Figure 4:**
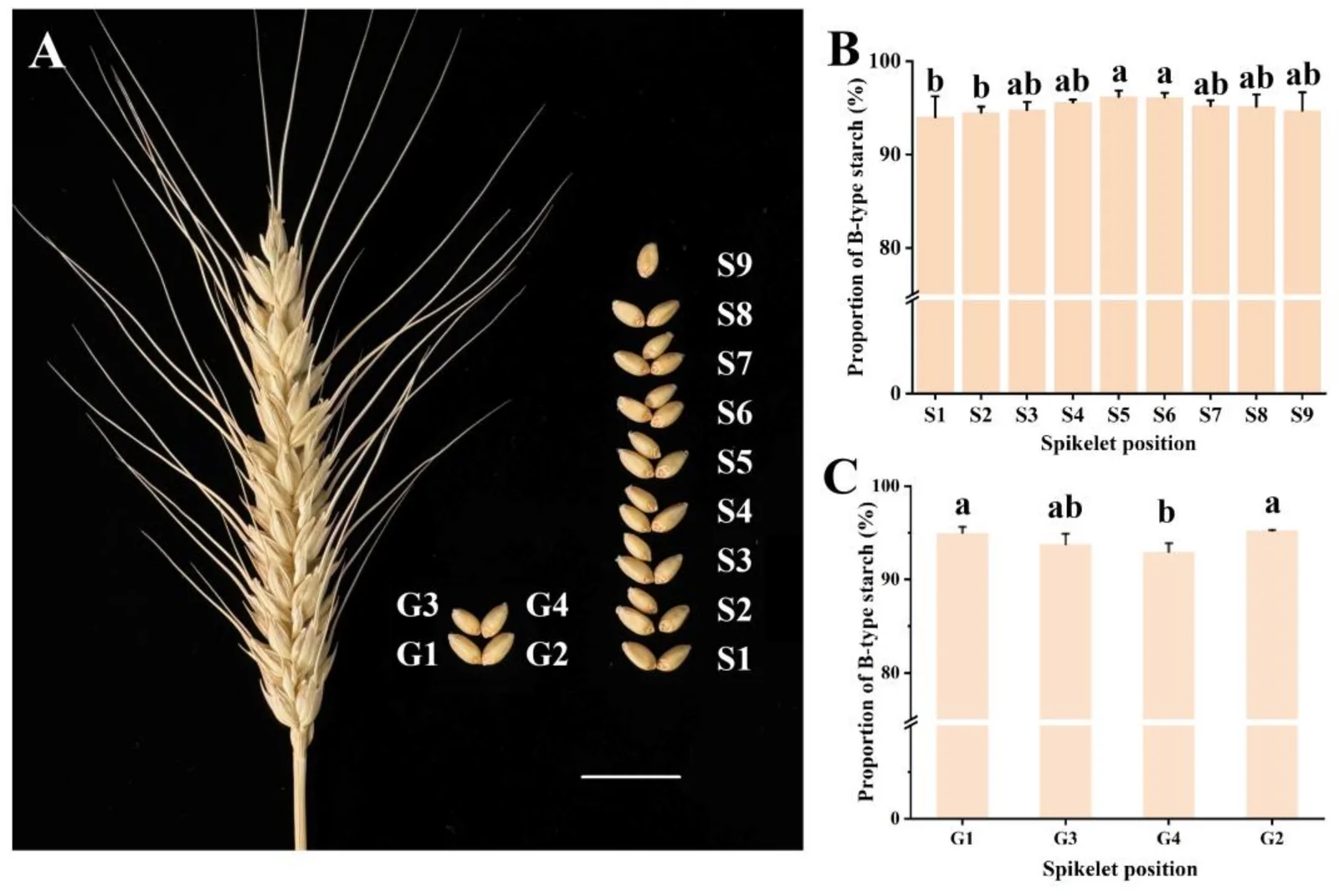
The starch granule distribution in superior and inferior grains. Scale bars = 2 cm. (**A**) Schematic representation of grain positions within a single wheat spike. S1–S9 indicate different spikelets from bottom to top within one spike. (**B**) Comparison of the B-type starch proportion across longitudinal positions within the spike (basal, middle, and apical). (**C**) Comparison of B-type starch proportion between lateral grains (G1 and G2) and central grains (G3 and G4) within individual spikelets. Data are presented as means ± SD of three biological replicates. Each biological replicate corresponds to one independent spike. At each designated position, one grain was sampled per spike and analyzed using 10 microscopic images. Different letters indicate significant differences among groups, as determined by one-way analysis of variance (ANOVA) followed by Tukey’s test (*p* < 0.05).

Beyond capturing the temporal dynamics of starch accumulation, the SSDSG method enables precise characterization of spatial variation in starch granule size distribution among individual grains occupying distinct positions within the same spike (Figure 4A). Marked differences in the proportion of B-type starch granules were observed among grain positions. Along the longitudinal axis of the spike, grains at the middle positions exhibited significantly higher proportions of B-type starch granules (96.17% for S5 and 96.10% for S6) than those at the basal (93.99% for S1 and 94.47% for S2) and apical positions (95.11% for S8 and 94.69% for S9) (Figure 4B). Similarly, within individual spikelets, lateral grains (94.99% for G1 and 95.24% for G2) consistently exhibited higher proportions of B-type starch granules than the central grains (93.78% for G3 and 92.95% for G4) (Figure 4C). These positional differences could partly reflect variations in assimilate partitioning and inter-grain competition during grain filling, as well as variation in the development and connectivity of vascular bundles and nutrient-transport tissues among grain positions [41]. However, these interpretations remain tentative, and further studies are needed to establish the underlying mechanisms. Such intra-spike heterogeneity would likely be obscured by conventional bulk-based analyses, highlighting the unique ability of the SSDSG approach to resolve biologically relevant variation at the single-grain level.

Collectively, these results demonstrate that the SSDSG method provides a versatile in situ, single-grain platform for characterizing starch granule development with minimal sample input. By enabling stage-specific assessment of starch granule phenotypes, the method offers a practical approach for the rapid functional evaluation of starch biosynthesis genes within a single generation, including in transgenic lines, mutants, and other non-bulked materials. For example, gene function of *SS4* could be evaluated during early grain development [37], when abnormalities in starch granule morphology are most pronounced, whereas regulators of B-type starch granule formation, including *MFP1* [42] and *PHS1* [40], could be characterized during the mid-to-late stages of grain filling, when B-type granule initiation is most active. Furthermore, the ability of SSDSG to resolve positional differences in B-type starch granule abundance provides a robust framework for investigating source–sink interactions in plants. Overall, this platform has considerable potential to accelerate the functional characterization of starch-related genes and to facilitate wheat quality improvement and high-throughput screening of large germplasm populations.

### 3.3. High-Throughput Screening and Characterization of Starch Size Distribution in an EMS-Mutagenized Wheat Population

Ethyl methanesulfonate (EMS) mutagenesis is an effective approach for generating diverse mutant populations in wheat and can induce a broad spectrum of quantitative phenotypic variations [35]. These variations provide valuable genetic resources for gene discovery and the functional characterization of agronomically important traits. Given the critical role of B-type starch granules in wheat quality, identifying mutant lines with altered B-type granule phenotypes is particularly valuable for elucidating the genetic and regulatory mechanisms underlying starch granule biogenesis, characterizing genes involved in starch metabolism, and facilitating the development of wheat varieties with improved quality traits [2].

However, the large size of mutant populations poses a major challenge for phenotypic screening, as conventional approaches are often labor-intensive and time-consuming. Conventional methods for analyzing starch granule size distribution generally require relatively large sample quantities and involve cumbersome, time-consuming procedures, resulting in substantial analytical costs. These limitations considerably restrict their applicability to large-scale screening of mutant populations. To address these bottlenecks, we employed the SSDSG method for high-throughput screening of starch granule size distribution. The method combines rapid analysis, practical analytical performance, and minimal sample requirements, with only a single wheat grain needed for each assay. This feature renders it exceptionally well-suited for screening valuable, rare, or limited mutant materials, while substantially improving the efficiency, throughput, and feasibility of large-scale phenotypic screening.

**Figure 5:**
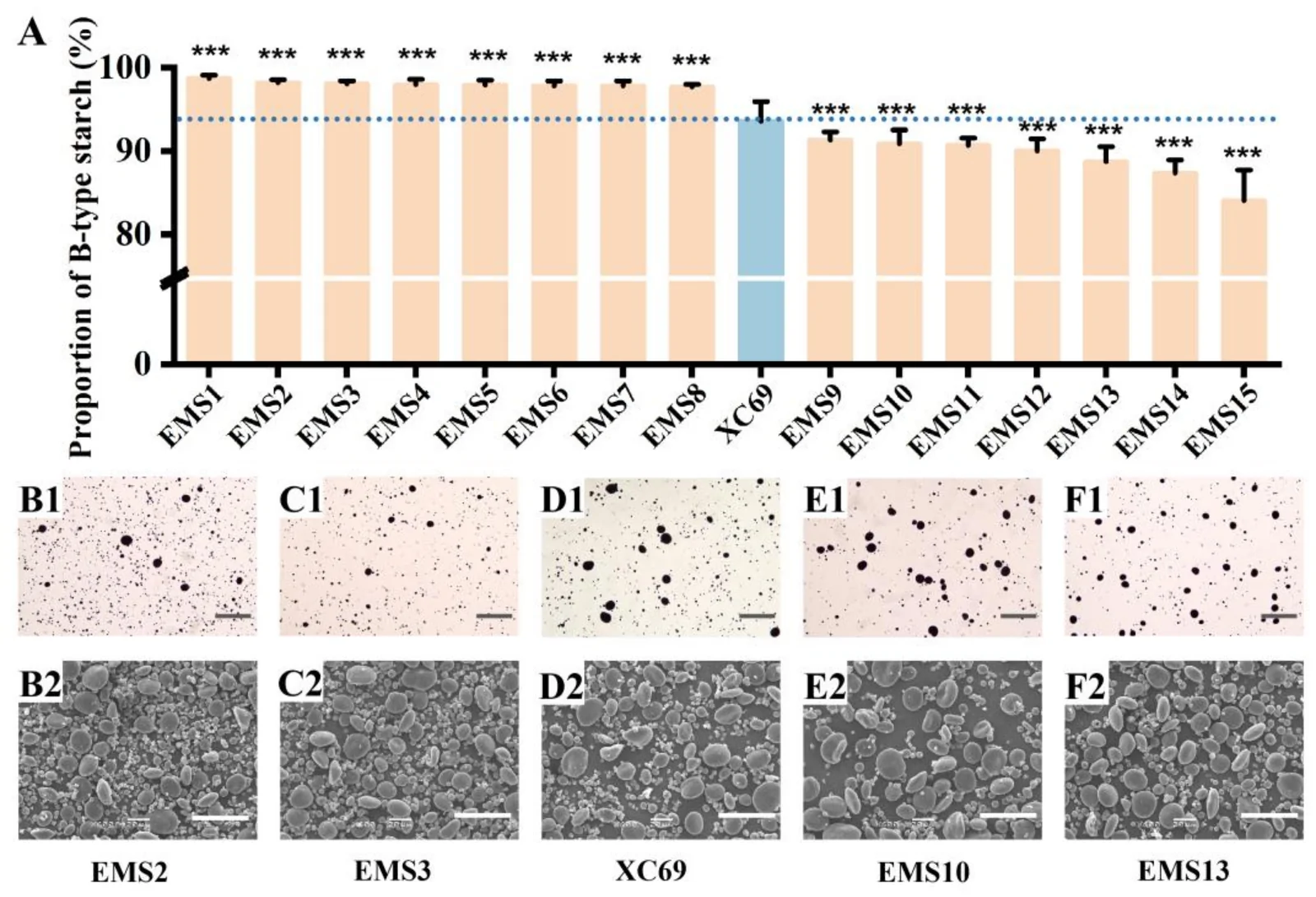
Screening and validation of the EMS-mutagenized wheat population for variations in B-type starch content. (**A**) Distribution of B-type starch proportions across the representative EMS-mutagenic lines. The B-type starch proportion of the non-mutagenized control XC69 is indicated by the blue bar and blue dashed line, while the orange bars represent that of the EMS-mutagenized lines. (**B1**–**F1**) Morphology of starch granules observed by electron microscopy. Scale bars = 50 μm. (**B2**–**F2**) Scanning Electron Microscope (SEM) micrographs of the selected mutant lines exhibiting extreme B-type starch phenotypes. Asterisks denote statistically significant differences between XC69 and the EMS-mutagenized wheat population based on Student’s *t*-test (*** *p* < 0.001).

To assess the practical applicability of the SSDSG method for large-scale screening, we established an EMS-mutagenized population comprising 1667 mutant lines derived from the wheat cultivar Xichang 69. High-throughput screening of this population identified 8 mutant lines with significantly increased B-type starch proportions (97.82–98.69%) and 7 lines with significantly decreased proportions (84.01–91.30%), compared with the non-mutagenized control XC69 (93.58%) (Figure 5A). The 15 mutants displaying extreme B-type starch phenotypes were subsequently examined by Scanning Electron Microscope (SEM), with representative micrographs presented in Figure 5B–F. SEM observations confirmed pronounced alterations in st arch granule distribution patterns within the endosperm of the selected mutants, consistent with the quantitative measurements obtained using SSDSG. The agreement between SSDSG-based screening and SEM observations demonstrates the reliability of SSDSG for efficiently identifying wheat mutants with altered starch granule characteristics at the single-grain level. It has been previously reported that Wx/GBSSI inactivation in some EMS-induced wheat mutants alters the proportion of B-type starch granules, though it is accompanied by reduced total starch content and largely unchanged granule morphology [43,44]. For the mutagenized materials generated in these experiments, our SSDSG method enabled high-throughput screening to identify extreme variants. Consequently, this high-throughput screening approach provides powerful technical support for investigating the genetic and developmental mechanisms underlying starch granule formation, gene mapping, candidate gene validation, and facilitating the development of germplasm resources for wheat quality improvement and trait-oriented breeding [45,46].

### 3.4. Non-Destructive Characterization of Starch Size Distribution Enables Generation Advancement in Early-Stage Breeding Materials

Wheat quality breeding is essential for improving grain end-use quality and enhancing the industrial value of wheat [47]. Starch composition and granule developmental characteristics are critical determinants of wheat processing performance and nutritional quality and therefore represent key targets for genetic improvement [4,48,49]. However, conventional quality breeding programs are often constrained by lengthy breeding cycles and limited selection efficiency. These challenges are particularly pronounced in early-generation populations, where large population sizes and limited seed quantities per individual plant make accurate and high-throughput phenotypic screening difficult. Recent advances in single-seed phenotyping have partially alleviated this bottleneck; for instance, integrated near-infrared and 3D-reconstruction platforms and single-kernel NIR sorting have enabled non-destructive, high-throughput prediction of protein content and breadmaking quality in early generations [50,51]. However, these emerging platforms have primarily focused on protein content, seed viability, or overall grain morphology. High-throughput, embryo-preserving tools specifically tailored for the quantitative characterization of starch granule size distribution remain scarce. As a result, the rapid selection and directional improvement of starch-related quality traits, as well as the efficient identification of elite germplasm, remain significant challenges in wheat quality breeding.

**Figure 6:**
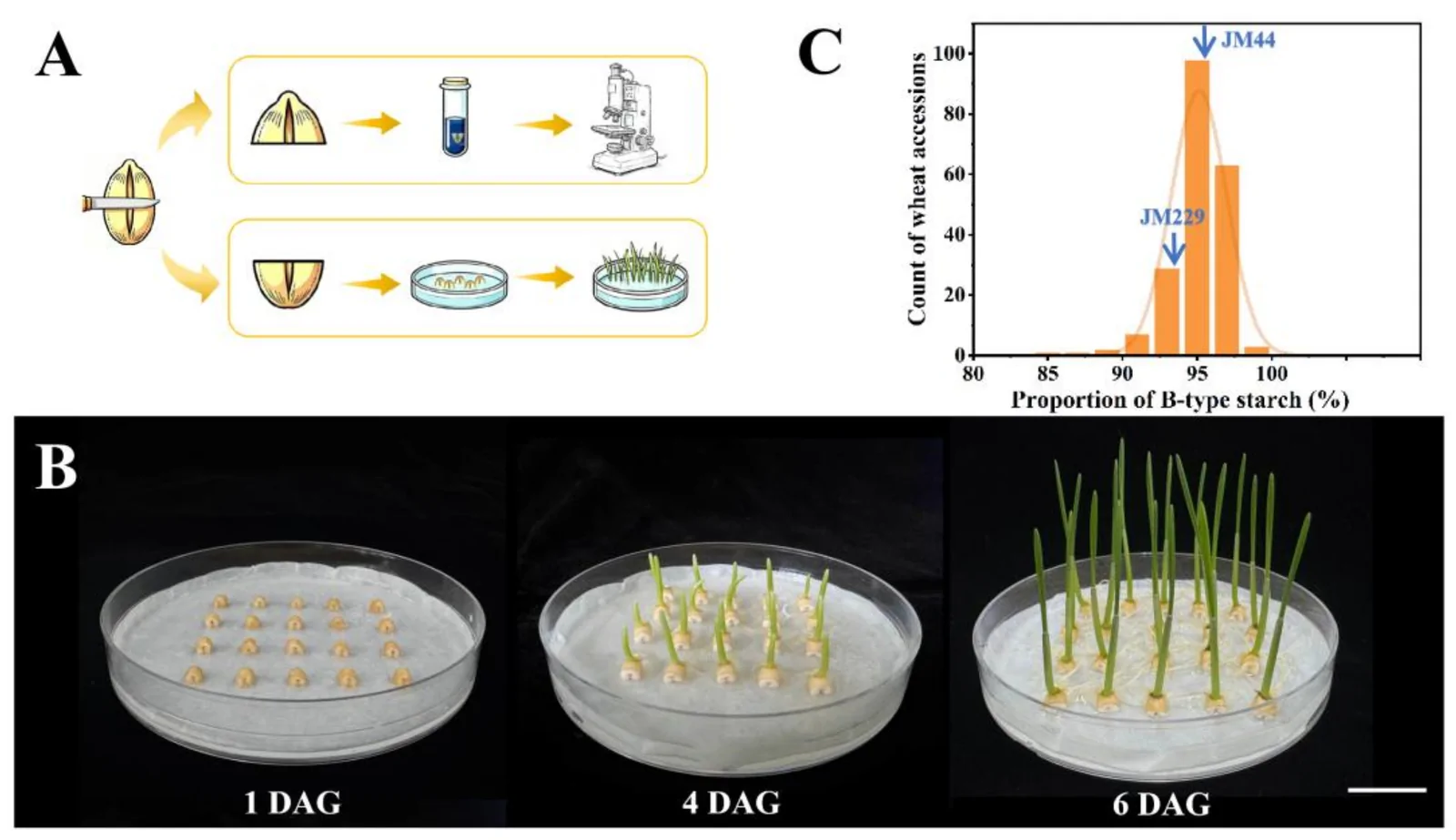
Non-destructive characterization of starch size distribution for advancing early-stage breeding generations. (**A**) Workflow for non-destructive phenotyping and single-grain screening. (**B**) Subculture of the corresponding embryo-retaining lower half following phenotypic screening. Scale bars = 2 cm. (**C**) Statistics of starch size distribution using the embryo-less half-seed across the 204 F_2_ breeding lines.

The SSDSG method established in this study is applicable not only to large-scale screening of EMS mutant populations but also to rapid phenotyping and targeted selection of early-generation wheat breeding materials. A key advantage of the method is its non-destructive, embryo-preserving workflow, which enables phenotypic evaluation and subsequent seed propagation from the same grain. As illustrated in Figure 6A, a single wheat grain is transversely sectioned at the equatorial midpoint. The upper half is used for precise characterization of starch granule size distribution, while the lower half, which retains the intact embryo, can be directly sown for generation advancement. Notably, the embryo-containing half-grain exhibited no significant adverse effects on germination or subsequent vegetative growth (Figure 6B), demonstrating that the sectioning procedure does not compromise seed viability. To evaluate the utility of SSDSG in early-generation breeding, we phenotyped starch granule size distribution in 204 F_2_ breeding lines. The top 10% of lines exhibiting extremely high B-type starch proportions can be subjected for further studying of their genetic stability, all of which also exceeded the B-type starch contents of their parental lines, Jimai44 (95.59%) and Jimai229 (93.68%) (Figure 6C). These selected materials remained viable for further propagation and selection, facilitating their incorporation into subsequent stages of high-quality wheat variety development. Collectively, the SSDSG method enables high-throughput, quantitative phenotyping of starch-related traits while simultaneously preserving the genetic material required for generation advancement. By integrating phenotyping, selection, and seed propagation within the same generation, the method substantially improves the efficiency of directional selection in early-generation breeding. This approach can shorten the breeding cycle and enhance selection efficiency for end-use quality traits, providing a robust technical platform for improving the efficiency of wheat breeding programs and accelerating the development of high-quality, high-yielding wheat varieties.

### 3.5. Cross-Species Characterization of Starch Granule Size Distribution in Diverse Cereals and Crop Species Using the SSDSG Method

**Figure 7:**
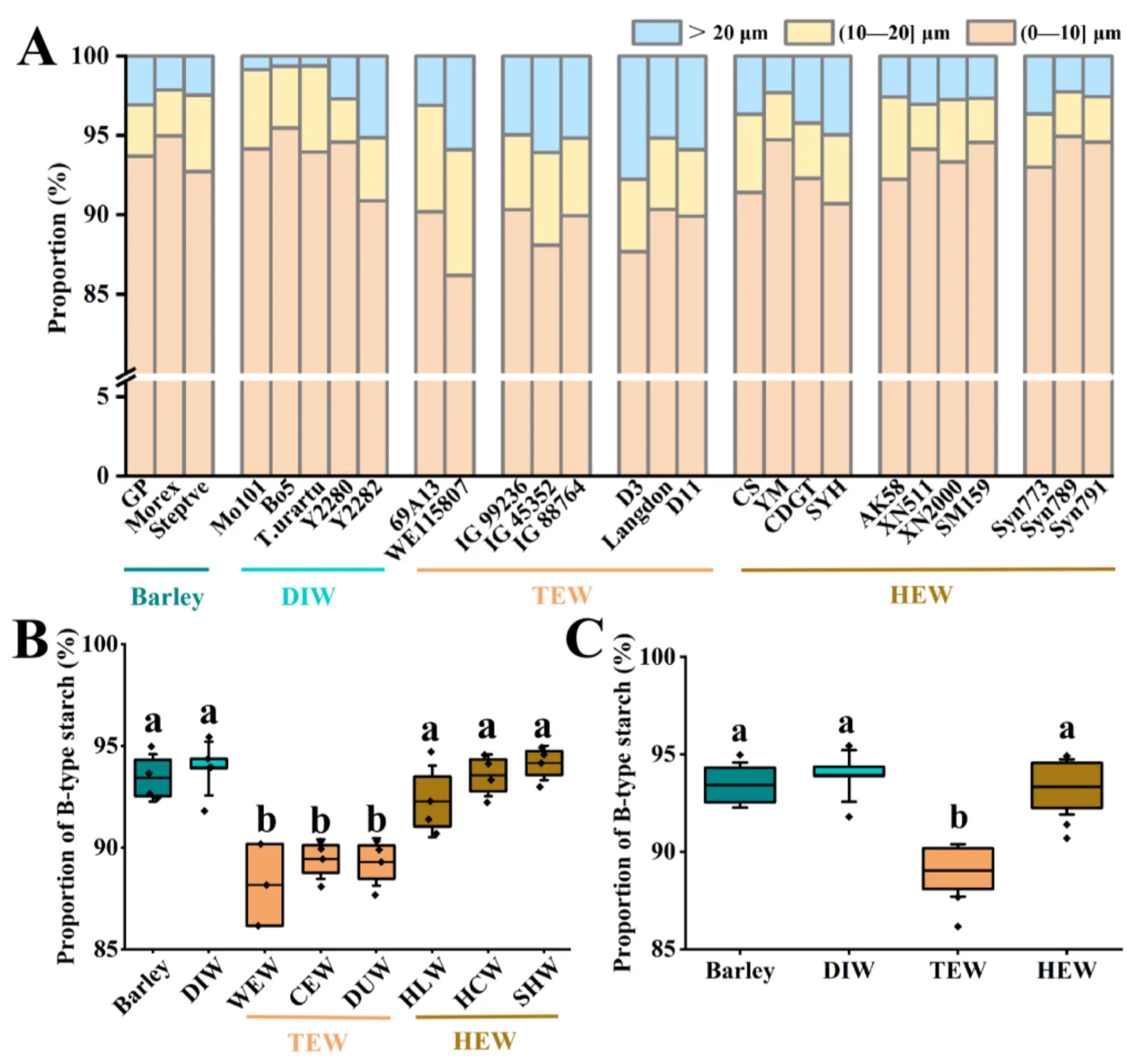
Application of SSDSG (Determination of starch size distribution in single grain) method to characterize starch size distribution across diverse barley and wheat species. DIW: Diploid wheat; TEW: Tetraploid wheat; HEW: Hexaploid wheat; WEW: Wild emmer wheat; CEW: Cultivated emmer wheat; DUW: Durum wheat; HLW: Hexaploid landrace wheat; HCW: Hexaploid cultivated wheat; SHW: Synthetic hexaploid wheat. (**A**) Comparative analysis of starch size distribution across 27 accessions representing different ploidy levels and genetic backgrounds of the test materials. (**B**) Comparative analysis of B-type starch proportion across diverse barley and wheat species. (**C**) Comparative analysis of B-type starch proportion across diverse ploidy materials. Error bars represent standard deviations from three biological replicates. Different letters indicate significant differences among groups, as determined by one-way analysis of variance (ANOVA) followed by Tukey’s test (*p* < 0.05).

Beyond its application to common wheat, the versatility of the SSDSG method was further evaluated by characterizing starch granule size distributions across diverse cereal species and wheat ploidy levels [52,53]. Unlike crops such as maize, buckwheat, rice, and potato that lack a specialized multiple-size distribution, wheat and barley exhibit a distinct bimodal size distribution in their starch granules. Therefore, barley and wheat accessions of different ploidy levels were selected specifically for this comparison to demonstrate the cross-species applicability of SSDSG. As shown in Figure 7A, barley and diploid, tetraploid, and hexaploid wheat exhibited distinct variations in the proportion of B-type starch granules. These ploidy-associated variations reflect the complex genetic regulation of B-type granules. As previously reported, tetraploid-*Aegilops sharonensis* amphidiploids exhibit intermediate A/B-type granule ratios relative to their parents, indicating that polyploidization can produce intermediate phenotypes rather than a simple linear trend [54]. These findings confirm that SSDSG captures the non-monotonic B-type variation across ploidy levels, demonstrating its value for efficient germplasm screening.

A comparison of the 27 wheat accessions is illustrated in Figure 7. No significant difference in B-type starch granule proportion was observed between barley and diploid wheat, which showed B-type proportions of 93.43% and 93.89%, respectively (Figure 7B). Tetraploid wheat exhibited a relatively lower proportion of B-type starch granule proportion of 89.04%, and no significant differences were detected among wild emmer, cultivated emmer, and durum wheat within the tetraploid group. In contrast, hexaploid wheat had a higher proportion of B-type starch granule proportion of 93.33% than tetraploid wheat, while its B-type starch granule proportion remained comparable to those of barley and diploid wheat (Figure 7C). Overall, these findings are consistent with previous reports documenting evolutionary changes in starch granule size distribution associated with ploidy level within the Triticeae [55,56].

These results demonstrate that the SSDSG method is applicable not only to common wheat but also to barley and other Triticeae species, enabling robust and high-throughput characterization of starch granule size distribution across species with diverse ploidy levels and genetic backgrounds. Notably, the method requires neither species-specific staining nor complex sample pretreatment, highlighting its broad cross-species applicability. This versatility suggests considerable potential for extending the SSDSG method to other cereal crops, such as rice, maize, and sorghum, as well as starch-rich tuber crops such as potato and cassava. As a high-throughput and cost-effective phenotyping method, SSDSG provides a robust technical platform to support starch quality improvement, germplasm screening, and comparative studies of the mechanisms underlying starch biosynthesis and spatial accumulation across diverse crop species.

Compared with conventional methods, SSDSG substantially improves both screening throughput and breeding efficiency in four key aspects, which can be potentially utilized in phenotyping analysis in early generations of breeding lines. First, in terms of time efficiency, the conventional workflow, including grinding, starch extraction, sedimentation, drying, sample preparation, and instrumental analysis, typically requires about 6 days, whereas the SSDSG workflow can be completed in less than 3 days. Second, regarding sample consumption, conventional methods typically require at least 6 g of starting material, whereas SSDSG requires only a single grain (approximately 30–50 mg), representing a reduction of more than 100-fold. Third, in terms of breeding efficiency, the embryo-preserving feature of SSDSG allows the same grain to be directly sown after phenotyping, thereby integrating selection and generation advancement within the same generation. In contrast, conventional methods destroy the grain during extraction, requiring evaluation to be delayed until the next generation. Finally, conventional grinding and drying steps are often impractical for developing grains during the filling stage due to their high moisture content. In contrast, SSDSG is directly applicable to developing grains, enabling real-time monitoring of starch granule development.

## 4. Conclusions

In conclusion, we developed a novel, high-throughput, imaging-based method, designated the SSDSG method, for the rapid and quantitative characterization of starch granule size distribution at the single-grain level in wheat. The method enables in situ, non-destructive phenotyping while addressing several key limitations associated with conventional techniques. By integrating a commercial imaging system with custom-developed HTDP software, SSDSG enables automated batch processing of microscopic images and generates comprehensive quantitative morphological parameters for individual starch granules.

It is worth noting that this imaging-based SSDSG approach has inherent field-of-view dependence. Fields of view should be selected in a completely random manner; peripheral regions as well as areas featuring severe granule aggregation and overlapping should be excluded from analysis. In addition, the morphological correction factor for A-type granules is determined based on an assumed oblate-spheroid geometry; further experimental validation of granule thickness and shape variation across genotypes is therefore warranted. Future work will focus on optimizing the morphological model and expanding the application scope of SSDSG to a wider range of cereal and tuber crops, as well as integrating it with genetic mapping and gene-editing pipelines to further improve throughput and robustness.

Overall, SSDSG provides a robust, high-throughput, and cost-effective phenotyping platform with substantial potential to accelerate starch-related genetic research and quality-oriented wheat breeding.

## Data Availability

The original contributions presented in this study are included in the article/Supplementary Materials. Further inquiries can be directed to the corresponding authors.

## Supplementary Materials

The following supporting information can be downloaded at: https://www.mdpi.com/article/10.3390/foods15213971/s1, Figure S1: Calculation of the average correction factor $\tilde{K}$; Table S1: The detailed calculation results of the factor K; Table S2: The detailed comparative results of the 20 wheat varieties analyzed; Source Code S1: Source Code of High-Throughput Data Processing Software Based on MATLAB Platform (Version R2022a).

## Abbreviations

The following abbreviations are used in this manuscript:

SSDSG	Determination of starch size distribution in single grains
SEM	Scanning electron microscopy
HTDP-software	High-throughput data processing software
EMS	Ethyl methanesulfonate
