# Supplementary material for: Determination of Starch Size Distribution in Single Grains (SSDSG): A Novel High-Efficiency Method for Characterizing Starch Granule Morphology in Wheat
Source: Foods. 2026 Nov 5;15(21):3971. doi: 10.3390/foods15213971 (PMC13763367; doi:10.3390/foods15213971)
Supplement: Supplementary file 1 [file foods-15-03971-s001.zip › foods-4633012-supplementary.pdf]

# Supplementary Material

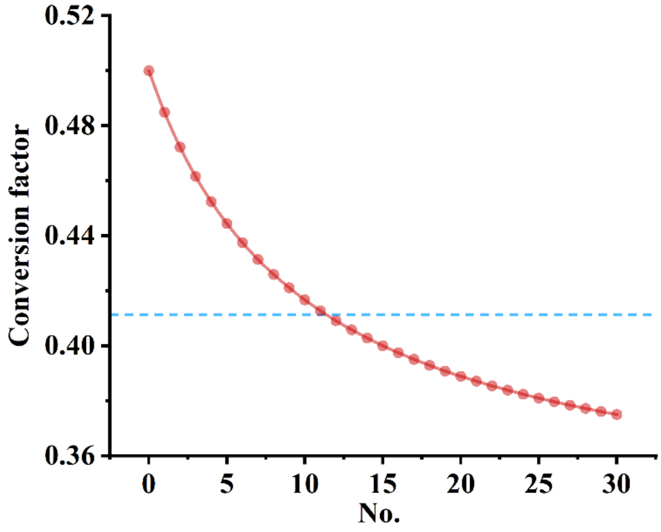


**Figure S1.** Calculation of the average correction factor $\widetilde{K}$.

**Table S1.** The detailed calculation results of the factor ***K***.

| No. | Thickness 2c (μm) | Diameter 2a (μm) | Conversion factor (K = c/a) |
|---|---|---|---|
| 0 | 5.0000 | 10.0000 | 0.5000 |
| 1 | 5.3333 | 11.0000 | 0.4848 |
| 2 | 5.6667 | 12.0000 | 0.4722 |
| 3 | 6.0000 | 13.0000 | 0.4615 |
| 4 | 6.3333 | 14.0000 | 0.4524 |
| 5 | 6.6667 | 15.0000 | 0.4444 |
| 6 | 7.0000 | 16.0000 | 0.4375 |
| 7 | 7.3333 | 17.0000 | 0.4314 |
| 8 | 7.6667 | 18.0000 | 0.4259 |
| 9 | 8.0000 | 19.0000 | 0.4211 |
| 10 | 8.3333 | 20.0000 | 0.4167 |
| 11 | 8.6667 | 21.0000 | 0.4127 |
| 12 | 9.0000 | 22.0000 | 0.4091 |
| 13 | 9.3333 | 23.0000 | 0.4058 |
| 14 | 9.6667 | 24.0000 | 0.4028 |
| 15 | 10.0000 | 25.0000 | 0.4000 |
| 16 | 10.3333 | 26.0000 | 0.3974 |
| 17 | 10.6667 | 27.0000 | 0.3951 |
| 18 | 11.0000 | 28.0000 | 0.3929 |
| 19 | 11.3333 | 29.0000 | 0.3908 |
| 20 | 11.6667 | 30.0000 | 0.3889 |
| 21 | 12.0000 | 31.0000 | 0.3871 |
| 22 | 12.3333 | 32.0000 | 0.3854 |
| 23 | 12.6667 | 33.0000 | 0.3838 |
| 24 | 13.0000 | 34.0000 | 0.3824 |
| 25 | 13.3333 | 35.0000 | 0.3810 |
| 26 | 13.6667 | 36.0000 | 0.3796 |
| 27 | 14.0000 | 37.0000 | 0.3784 |
| 28 | 14.3333 | 38.0000 | 0.3772 |
| 29 | 14.6667 | 39.0000 | 0.3761 |
| 30 | 15.0000 | 40.0000 | 0.3750 |

**Table S2.** Detailed comparative results of the 20 wheat varieties analyzed.

| Variety | Volume proportion of B-type starch (%) | Volume proportion of A-type starch (%) | Transformed volume proportion of B-type starch (%) | Transformed volume proportion of A-type starch (%) |
|---|---|---|---|---|
| Nongda8987 | 34.97 | 65.03 | 41.87 | 58.13 |
| Qifeng2 | 34.00 | 66.00 | 44.84 | 55.16 |
| Daimai3166 | 33.72 | 66.28 | 34.92 | 65.08 |
| Shannong0431 | 33.40 | 66.60 | 47.22 | 52.78 |
| Zhoumai40 | 32.35 | 67.65 | 39.53 | 60.47 |
| JinongCH01 | 32.15 | 67.85 | 31.32 | 68.68 |
| Xinong979 | 32.06 | 67.94 | 34.30 | 65.70 |
| JimaiU80 | 32.01 | 68.00 | 38.48 | 61.52 |
| Xingmai13 | 31.90 | 68.10 | 31.02 | 68.98 |
| Shannong25 | 31.77 | 68.24 | 35.29 | 64.71 |
| Hengguan35 | 30.69 | 69.32 | 21.56 | 78.44 |
| Hongdi88 | 30.52 | 69.48 | 43.51 | 56.49 |
| Jihe12339 | 29.74 | 70.26 | 34.33 | 65.67 |
| Huaimai35 | 29.47 | 70.53 | 35.64 | 64.36 |
| Yannong187 | 28.64 | 71.36 | 28.50 | 71.50 |
| XumaiDH9 | 28.32 | 71.68 | 23.00 | 77.00 |
| Zhongyu1526 | 27.60 | 72.40 | 23.67 | 76.33 |
| Zhoumai32 | 26.95 | 73.05 | 22.02 | 77.98 |
| Shimai27 | 26.69 | 73.31 | 18.26 | 81.74 |
| Zhoumai38 | 23.33 | 76.67 | 24.29 | 75.71 |

**Source Code S1.** Source Code of High-Throughput Data Processing Software Based on MATLAB Platform.

```
//-----------------------------------------------------------------------------
//main.cpp
#include <stdio.h>
#include <stdlib.h>
#include <fstream>
#include <iostream>
#include <direct.h> // include getcwd function declaration
#include <limits.h> // include PATH_MAX declaration
#include <windows.h> // include PATH_MAX declaration for windows
#include <string>
#include <vector>
#include <iomanip>
#include <cmath>

using namespace std;

string data_dir, prefix;
int SAMPLES, N;
vector<string> dir;
vector<vector<string>> filelist;
vector<vector<string>> datas;
vector<string> viewlist, yplist;
vector<int> cnts;
int maxCnt, totalCnt;

void read_ini();
void readlist();
void extract();
void extracta();
void stata(int n);
void writeall();
void writeall1();
void readoneview(vector<string>& vfln);
void writeonesample(int n);
vector<string> string_split(const string& s, char delimiter);
vector<vector<double>> stat(vector<vector<string>>& data, int no, int total_num, int&
ANum);

struct clsAB {
   int ANum, BNum, Total;
   double APerc, BPerc;
};
```

```
vector <clsAB> listab;
struct clsAll {
    string Name;
    int ANum, BNum, Total;
    double APerc, BPerc;
    double APercMean, BPercMean;
    double APercSDev, BPercSDev;
};
vector<clsAll> all, all1;
vector<int> pzFreq;
int pzTotalCnt;

int main() {
    read_ini();
    //printf("Data folder: %s\nfile name prefix: %s\nnumber of samples: %d\nnumber of
views: %d\n", data_dir.c_str(), prefix.c_str(), N, SAMPLES);
    printf("Data folder: %s\n", data_dir.c_str());
    readlist();
    //extract();
    extracta();
    writeall();
    writeall1();
    getchar();
}
void writeall1() {
    string fileo = data_dir + "\\output\\all-1.csv";
    std::cout << "Write to: " << fileo << endl;
    ofstream file(fileo);
    string line("Name,APercMean,BPercMean,APercSDev,BPercSDev");
    file << line << endl;
    int i0 = 0, i1, num;
    double APercMean = 0, BPercMean = 0, APercSDev = 0, BPercSDev = 0;
    vector<string> tokens = string_split(all[0].Name, '-');
    string prepinzh = tokens[0], pinzh;
    file << setprecision(6);
    for (int i = 0; i < all.size(); i++) {
        vector<string> tokens = string_split(all[i].Name, '-');
        pinzh = tokens[0];
        if (pinzh != prepinzh) {
            i1 = i;
            num = i1 - i0;
            APercMean /= num;
            BPercMean /= num;
            APercSDev = 0;
```

```
        BPercSDev = 0;
        for (int j = i0; j < i1; j++) {
            APercSDev += (all[j].APercMean - APercMean) * (all[j].APercMean - APercMean);
            BPercSDev += (all[j].BPercMean - BPercMean) * (all[j].BPercMean - BPercMean);
        }
        APercSDev = sqrt(APercSDev / (num - 1));
        BPercSDev = sqrt(BPercSDev / (num - 1));
        file << prepinzh << ",";
        file << APercMean << "," << BPercMean << ",";
        file << APercSDev << "," << BPercSDev << endl;

        prepinzh = pinzh;
        i0 = i;
        APercMean = 0;
        BPercMean = 0;
    }
    APercMean += all[i].APercMean;
    BPercMean += all[i].BPercMean;
  }

  //the last pinzh
  i1 = all.size();
  num = i1 - i0;
  APercMean /= num;
  BPercMean /= num;
  APercSDev = 0;
  BPercSDev = 0;
  for (int j = i0; j < i1; j++) {
    APercSDev += (all[j].APercMean - APercMean) * (all[j].APercMean - APercMean);
    BPercSDev += (all[j].BPercMean - BPercMean) * (all[j].BPercMean - BPercMean);
  }
  APercSDev = sqrt(APercSDev / (num - 1));
  BPercSDev = sqrt(BPercSDev / (num - 1));
  file << prepinzh << ",";
  file << APercMean << "," << BPercMean << ",";
  file << APercSDev << "," << BPercSDev << endl;

  file.close();
}
void writeall() {
  string fileo = data_dir + "\\output\\all.csv";
  std::cout << "Write to: " << fileo << endl;
  ofstream file(fileo);
  string
```

```
line("Name,ANum,BNum,Total,APerc,BPerc,APercMean,BPercMean,APercSDev,BPercS
Dev");
    file << line << endl;
    for (int i = 0; i < all.size(); i++) {
        file << all[i].Name << ",";
        file << all[i].ANum << "," << all[i].BNum << "," << all[i].Total << ",";
        file << setprecision(6) << all[i].APerc << "," << all[i].BPerc << ",";
        file << all[i].APercMean << "," << all[i].BPercMean << ",";
        file << all[i].APercSDev << "," << all[i].BPercSDev << endl;
    }
    file.close();
}
void writepzFreq(int n) {
    int fmax = pzFreq.size();
    vector<int> freq_a(fmax, 0);
    freq_a[0] = pzFreq[0];
    for (int i = 1; i < fmax; i++)
    {
        freq_a[i] = freq_a[i - 1] + pzFreq[i];
    }
    string fileo = data_dir + "\\output\\" + filelist[n][0] + "-" + "freq.csv";
    std::cout << "Write to: " << fileo << endl << endl;
    ofstream file(fileo);
    string line("d,f,Cnt,aCnt,Freq,aFreq");
    file << line << endl;
    double d = 0.5;
    double p, pa;
    int k = 1;
    for (int i = 0; i < fmax; i++)
    {
        file << fixed << setprecision(1) << d << ",";
        d += 1;
        file << k++ << "," << pzFreq[i] << "," << freq_a[i] << ",";
        p = (double)pzFreq[i] / pzTotalCnt;
        pa = (double)freq_a[i] / pzTotalCnt;
        file << fixed << setprecision(6) << p << "," << pa << endl;
    }
    file.close();

}
void stata(int n)
{
    writeonesample(n);
    vector<vector<double>> data_d;
```

```
double d, dmax = 0;
//convert to double
for (auto& adatas : datas)
{
    vector<double> adata_d;
    for (auto& s : adatas)
    {
        d = stod(s);
        adata_d.push_back(d);
        if (d > dmax)
            dmax = d;
    }
    data_d.push_back(adata_d);
}
int fmax = ceil(dmax);
vector<int> freq(fmax, 0);
for (auto& d_r : data_d)
{
    for (auto& d : d_r)
        freq[(int)d]++;
}
////categories
if (pzFreq.size() == 0) {
    pzFreq = freq;
    pzTotalCnt = totalCnt;
}
else {
    for (int i = pzFreq.size(); i < freq.size(); i++)
        pzFreq.push_back(0);
    for (int i = 0; i < freq.size(); i++)
        pzFreq[i] += freq[i];
    pzTotalCnt += totalCnt;
}
////
vector<int> freq_a(fmax, 0);
freq_a[0] = freq[0];
//int ANum = freq[0];
for (int i = 1; i < fmax; i++)
{
    freq_a[i] = freq_a[i - 1] + freq[i];
    //if (i <= 10)
    //    ANum += freq[i];
}
string fileo = data_dir + "\\output\\" + filelist[n][0] + "-" + filelist[n][1] + "freq.csv";
```

```
std::cout << "Write to: " << fileo << endl;
ofstream file(fileo);
string line("d,f,Cnt,aCnt,Freq,aFreq");
file << line << endl;
d = 0.5;
double p, pa;
int k = 1;
for (int i = 0; i < fmax; i++)
{
    file << fixed << setprecision(1) << d << ",";
    d += 1;
    file << k++ << "," << freq[i] << "," << freq_a[i] << ",";
    p = (double)freq[i] / totalCnt;
    pa = (double)freq_a[i] / totalCnt;
    file << fixed << setprecision(6) << p << "," << pa << endl;
}
file.close();

//Count A and B
vector<clsAB> statAB;
int AANum = 0, BBNum = 0;
double APerc = 0, BPerc = 0;
int m = data_d.size();
for (vector<double>& adata_d : data_d) {
    int ANum = 0, BNum = 0;
    for (double d : adata_d) {
        if (d >= 10)
            ANum++;
        else
            BNum++;
    }
    AANum += ANum;
    BBNum += BNum;
    clsAB ab;
    ab.ANum = ANum;
    ab.BNum = BNum;
    ab.Total = ANum + BNum;
    ab.APerc = (double)ANum / ab.Total;
    ab.BPerc = (double)BNum / ab.Total;
    APerc += ab.APerc;
    BPerc += ab.BPerc;
    statAB.push_back(ab);
}
clsAB tab;
```

```
    tab.ANum = AANum;
    tab.BNum = BBNum;
    tab.Total = AANum + BBNum;
    tab.APerc = (double)AANum / tab.Total;
    tab.BPerc = (double)BBNum / tab.Total;
    clsAB mab;
    mab.ANum = round((double)AANum / m);
    mab.BNum = round((double)BBNum / m);
    mab.Total = round((double)tab.Total / m);
    mab.APerc = APerc / m;
    mab.BPerc = BPerc / m;
    listab.push_back(mab);
    clsAB sdab;
    sdab.ANum = 0;
    sdab.BNum = 0;
    sdab.Total = 0;
    sdab.APerc = 0;
    sdab.BPerc = 0;
    for (auto& xab : statAB) {
        sdab.ANum += (xab.ANum - mab.ANum) * (xab.ANum - mab.ANum);
        sdab.BNum += (xab.BNum - mab.BNum) * (xab.BNum - mab.BNum);
        sdab.Total += (xab.Total - mab.Total) * (xab.Total - mab.Total);
        sdab.APerc += (xab.APerc - mab.APerc) * (xab.APerc - mab.APerc);
        sdab.BPerc += (xab.BPerc - mab.BPerc) * (xab.BPerc - mab.BPerc);
    }
    sdab.ANum = round(sqrt((double)sdab.ANum / (m - 1)));
    sdab.BNum = round(sqrt((double)sdab.BNum / (m - 1)));
    sdab.Total = round(sqrt((double)sdab.Total / (m - 1)));
    sdab.APerc = sqrt((double)sdab.APerc / (m - 1));
    sdab.BPerc = sqrt((double)sdab.BPerc / (m - 1));
    statAB.push_back(tab);
    statAB.push_back(mab);
    statAB.push_back(sdab);
    viewlist.push_back("Total");
    viewlist.push_back("Mean");
    viewlist.push_back("SDev");
    fileo = data_dir + "\\output\\" + filelist[n][0] + "-" + filelist[n][1] + "stat.csv";
    std::cout << "Write to: " << fileo << endl << endl;
    ofstream fileab(fileo);
    string lineab("View,ANum,BNum,TotalNum,APerc,BPerc");
    fileab << lineab << endl;
    for (int i = 0; i < m + 3; i++) {
        fileab << viewlist[i] << "," << statAB[i].ANum << "," << statAB[i].BNum << "," <<
statAB[i].Total << ",";
```

```
        fileab << setprecision(6) << statAB[i].APerc << "," << statAB[i].BPerc << endl;
    }
    file.close();
    clsAll a_all;
    a_all.Name = filelist[n][0] + "-" + filelist[n][1];
    a_all.ANum = tab.ANum;
    a_all.BNum = tab.BNum;
    a_all.Total = tab.Total;
    a_all.APerc = tab.APerc;
    a_all.BPerc = tab.BPerc;
    a_all.APercMean = mab.APerc;
    a_all.BPercMean = mab.BPerc;
    a_all.APercSDev = sdab.APerc;
    a_all.BPercSDev = sdab.BPerc;
    all.push_back(a_all);
}

void writeonesample(int n)
{
    cnts.resize(0);
    maxCnt = 0;
    totalCnt = 0;
    int k;
    for (int i = 0; i < datas.size(); i++) {
        k = datas[i].size();
        cnts.push_back(k);
        if (k > maxCnt)
            maxCnt = k;
        totalCnt += k;
    }
    string fileo = data_dir + "\\output\\" + filelist[n][0] + "-" + filelist[n][1] + ".csv";
    std::cout << "Write to: " << fileo << endl;
    ofstream file(fileo);
    string line("No");
    for (int j = 0; j < cnts.size(); j++)
    {
        line += ",";
        line += to_string(cnts[j]);
    }
    file << line << endl;
    int no = 1;
    for (int j = 0; j < maxCnt; j++)
    {
        line = to_string(no++);
```

```
        for (int k = 0; k < cnts.size(); k++)
        {
            line += ",";
            if (j < cnts[k])
                line += datas[k][j];
        }
        file << line << endl;
    }
    file.close();
}

void extracta()
{
    string prevpinzh, pinzh, prevyp, yp; // category, sample
    for (int i = 0; i < filelist.size(); i++)
    {
        pinzh = filelist[i][0];
        if (pinzh != prevpinzh) {
            if (prevpinzh != "") {
                stata(i - 1);
                writepzFreq(i - 1);
            }
            pzTotalCnt = 0;
            pzFreq.resize(0);
            prevpinzh = pinzh;
            prevyp = filelist[i][1];
            //cout << pinzh << endl;
            datas.resize(0);
            viewlist.resize(0);
            viewlist.push_back(filelist[i][2]);
            yplist.resize(0);
            yplist.push_back(filelist[i][1]);
            readoneview(filelist[i]);
        }
        else {
            yp = filelist[i][1];
            if (prevyp != yp)
            {
                stata(i - 1);
                prevyp = yp;
                datas.resize(0);
                viewlist.resize(0);
            }
            viewlist.push_back(filelist[i][2]);
```

```
            yplist.push_back(filelist[i][1]);
            readoneview(filelist[i]);
        }
    }
    stata(filelist.size() - 1);
    writepzFreq(filelist.size() - 1);
}
void readoneview(vector<string>& vfln)
{
    string fln = data_dir + "\\" + vfln[0] + "-" + vfln[1] + "-" + vfln[2] + ".csv";
    ifstream file(fln);
    string line;
    for (int k = 0; k < 2; k++)
    {
        getline(file, line);
        //cout << k << ":" << line << endl;
    }
    vector<string> data1;
    while (getline(file, line))
    {
        vector<string> tokens = string_split(line, ',');
        data1.push_back(tokens[3]);
    }
    file.close();
    datas.push_back(data1);
}
void readlist()
{
    string fln = data_dir + "\\dir.csv";
    cout << "Read from: "  << fln << endl;
    ifstream file(fln);
    string line;
    //getline(file, line);
    //cout << line << endl;
    //getline(file, line);
    //cout << line << endl;
    //cout << "Total number of data files: ";
    //vector<vector<string>> filelist;
    int i = 0;
    while (getline(file, line))
    {
        dir.push_back(line);
        vector<string> tokens = string_split(line, '-');
        if (tokens.size() != 3) {
```

```
            cout << "File format error: " << line << endl;
            exit(111);
        }
        filelist.push_back(tokens);
        tokens = string_split(tokens[2], '.');
        filelist[i][2] = tokens[0];
        i++;
    }
    file.close();
    N = filelist.size();
    cout << "Total number of data files: " << N << endl << endl;
}
void extract()
{
    string file = data_dir + "\\" + prefix;
    string filea, fileo;
    string fileNumFln = data_dir + "\\output\\" + prefix + "AB.csv";
    ofstream fileNum(fileNumFln);
    string s = "No,TotalNum,ANum,BNum,APerc,BPerc";
    fileNum << s << endl;
    int ANum = 0, BNum = 0;
    double APerc, BPerc;
    for (int i = 1; i <= SAMPLES; i++)
    {
        vector<vector<string>> data;
        for (int j = 1; j <= N; j++)
        {
            filea = file + to_string(i) + "-" + to_string(j) + ".csv";
            std::cout << "Read from: " << filea << endl;
            ifstream file(filea);
            string line;
            for (int k = 0; k < 2; k++)
            {
                getline(file, line);
                //cout << k << ":" << line << endl;
            }
            int k = 0;
            vector<string> data1;
            while (getline(file, line))
            {
                vector<string> tokens = string_split(line, ',');
                data1.push_back(tokens[3]);
            }
            file.close();
```

```
    data.push_back(data1);
}
vector<int> cnt;
int m = 0, total_num = 0;
for (int j = 0; j < N; j++)
{
    int k = data[j].size();
    cnt.push_back(k);
    if (k > m)
        m = k;
    total_num += k;
}
fileo = data_dir + "\\output\\" + prefix + to_string(i) + ".csv";
std::cout << "Write to: " << fileo << endl;
ofstream file(fileo);
string line("No,");
for (int j = 0; j < N; j++)
{
    line += to_string(cnt[j]);
    if (j < N - 1)
        line += ",";
}
file << line << endl;
int no = 1;
for (int j = 0; j < m; j++)
{
    line = to_string(no++) + ",";
    for (int k = 0; k < N; k++)
    {
        if (j < cnt[k])
            line += data[k][j];
        if (k < N - 1)
            line += ",";
    }
    file << line << endl;
}
file.close();
//////////////////////////////
auto data_d = stat(data, i, total_num, ANum);
BNum = total_num - ANum;
APerc = (double)ANum / total_num;
BPerc = 1 - APerc;
fileNum << i << "," << total_num << "," << ANum << "," << BNum << ",";
fileNum << fixed << setprecision(6) << APerc << "," << BPerc << endl;
```

```
    }
    fileNum.close();
    std::cout << "Write to：" << fileNumFln << endl;
}
vector<vector<double>> stat(vector<vector<string>> &data, int no, int total_num, int &ANum)
{
    vector<vector<double>> data_d;
    double d, dmax = 0;
    for (auto& adata : data)
    {
        vector<double> adata_d;
        for (auto& s : adata)
        {
            d = stod(s);
            adata_d.push_back(d);
            if (d > dmax)
                dmax = d;
        }
        data_d.push_back(adata_d);
    }
    int fmax = ceil(dmax);
    vector<int> freq(fmax, 0);
    for (auto& d_r : data_d)
    {
        for (auto& d : d_r)
            freq[(int)d]++;
    }
    vector<int> freq_a(fmax, 0);
    freq_a[0] = freq[0];
    ANum = freq[0];
    for (int i = 1; i < fmax; i++)
    {
        freq_a[i] = freq_a[i - 1] + freq[i];
        if (i <= 10)
            ANum += freq[i];
    }
    string fileo = data_dir + "\\output\\" + prefix + to_string(no) + "freq.csv";
    std::cout << "Write to: " << fileo << endl << endl;
    ofstream file(fileo);
    string line("d,f,Cnt,aCnt,Freq,aFreq");
    file << line << endl;
    d = 0.5;
    double p, pa;
```

```
    int k = 1;
    for (int i = 0; i < fmax; i++)
    {
        file << fixed << setprecision(1) << d << ",";
        d += 1;
        file << k++ << "," << freq[i] << "," << freq_a[i] << ",";
        p = (double)freq[i] / total_num;
        pa = (double)freq_a[i] / total_num;
        file << fixed << setprecision(6) << p << "," << pa << endl;
    }
    file.close();
    return data_d;
}

//--------------------------------------------------------------------------------
//read_ini.cpp
#include <fstream>
#include <iostream>
#include <string>

using namespace std;
extern string data_dir, prefix;
extern int SAMPLES, N;

void read_ini()
{
    ifstream file("data.ini");
    getline(file, data_dir);
    //getline(file, prefix);
    //file >> SAMPLES;
    //file >> N;
    file.close();
}
//--------------------------------------------------------------------------------
//string_split.cpp
#include <iostream>
#include <sstream>
#include <string>
#include <vector>

std::vector<std::string> string_split(const std::string& s, char delimiter) {
    std::vector<std::string> tokens;
    std::string token;
    std::istringstream tokenStream(s);
```

```
    while (std::getline(tokenStream, token, delimiter)) {
        tokens.push_back(token);
    }
    return tokens;
}
//-----------------------------------------------------------------------------
//data.ini
F:\data\XC69
```
